# *ASPM* May be Related to the Malignant Progression of Hepatitis B and is Associated With a Poor Prognosis of Hepatocellular Carcinoma

**DOI:** 10.3389/fbinf.2022.871027

**Published:** 2022-03-18

**Authors:** Siyou Tan, Wenyan Chen, Gaoyin Kong, Lai Wei

**Affiliations:** Clinical Research Center for Anesthesiology of ERAS in Hunan Province, Hunan Provincial People’s Hospital (The First Affiliated Hospital of Hunan Normal University), Changsha, China

**Keywords:** hepatocellular carcinoma (HCC), hepatitis B virus, bioinformatics analysis, ASPM, biomarker

## Abstract

**Background:** Hepatitis B virus (HBV) is a causative agent of hepatocellular carcinoma (HCC). Until now, the mechanism behind the progress of hepatitis B fibrosis to HCC remains largely unknown. This study aims to examine the candidate biomarkers and pathways involved in HBV-associated HCC.

**Methods:** Gene expression profiles were retrieved from the Gene Expression Omnibus (GEO) database. Differentially expressed genes (DEGs) were identified using the GEO2R tool after which functional enrichment analysis, protein-protein interaction (PPI) analysis, genetic alteration analysis, prognostic analysis, immune infiltration analysis, co-expression genes prediction, and miRNA-gene network construction, and pathway correlation analysis were performed.

**Results:** 22 hub genes were identified, which were all highly expressed in HCC, and overexpression of these genes was all associated with significantly worse survival in HCC patients. More significantly, *ASPM* also showed increased expression levels in non-tumor tissues with advanced liver fibrosis. With the progression of liver fibrosis and the closer tumor center of HCC, the higher expression of *ASPM* was identified. *ASPM* was considered to be the most promising biomarker because it also showed the highest genetic alteration frequency among the hub genes and the expression level of *ASPM* in HBV (+) HCC tissues was significantly higher than that in HBV (-) HCC tissues. Also, the infiltration levels of B cells, CD8^+^ T cells, CD4^+^ T cells, macrophages, neutrophils, and dendritic cells were all positively correlated with the expression of *ASPM*.

**Conclusion:** These findings may help in the development of strategies and candidate drugs for the treatment of HBV-related HCC and improve the effectiveness of personalized treatment in the future. *ASPM* was upregulated in both hepatitis B cirrhosis and HCC and could be a potential predicting biomarker.

## Introduction

**FIGURE 1:**
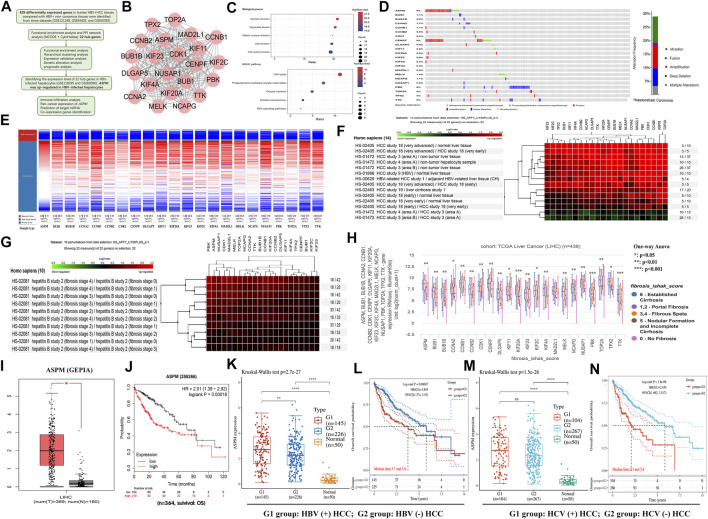
**(A)** Workflow of the present study. **(B)** The PPI network of 22 hub genes was performed by the STRING tool. **(C)** Biological process and KEGG pathway analysis of *ASPM*. **(D)** The genetic alteration analysis of 22 hub genes was performed by the cBioportal database **(E)** Hierarchical clustering of *ASPM* was constructed based on the UCSC database. **(F)** A Heatmap of *ASPM* expression in HCC tissues was constructed using GENEVESTIGATOR software. **(G)** A Heatmap of *ASPM* expression in liver fibrosis tissues was constructed using GENEVESTIGATOR software. **(H)** Box plot of ASPM mRNA expression in different stages of liver fibrosis tissues was created by UCSC online tool evaluated by the one-way Anova test **(I)** *ASPM* expression in liver tumor and normal people were verified using GEPIA online tool. **(J)** Overall survival analysis of HCC patients with different *ASPM* expression levels was performed using the Kaplan Meier plotter database. **(K)** *ASPM* expression in HBV (+) HCC, HBV (-) HCC, and adjacent non-tumor tissues were explored using the TCGA database. **(L)** Overall survival analysis of HBV (+) HCC patients compared with HBV (-) HCC patients was performed using the TCGA database. **(M)** *ASPM* expression in HCV (+) HCC, HCV (-) HCC, and adjacent non-tumor tissues were explored using the TCGA database. **(N)** TCGA database was used to analyze the overall survival of HCV (+) HCC patients and HCV (-) HCC patients. *: *p*-value < 0.05; **: *p*-value <0.01; ***: *p*-value <0.001.

*ASPM*, namely the abnormal spindle-like microcephaly-associated gene, is the human ortholog of the *Drosophila melanogaster* “abnormal spindleˮ gene (*ASP*) and was involved in the regulation of mitotic spindles and the coordination of mitotic processes (Wu et al., 2021). In humans, neurogenic defects that occur as a consequence of homozygous mutations in *ASPM* results in intellectual disability and microcephaly. Evidence indicated that *ASPM*, which is in the spindle poles, centrosomes, and midbodies, promotes cytokinesis and proliferative abilities of transformed human cell lines, human cancer cells, as well as fetal tissues (Wu et al., 2021). Researchers have found that *ASPM* acts as a novel regulator in promoting stemness by augmenting Wnt-Dvl-3-β-catenin signaling, and activation of Wnt signaling in cancer stem cells contributes to cancer progression in malignant tumors (Pai et al., 2019). Also, recent studies have shown that the expression level of *ASPM* in hepatocellular carcinoma (HCC) patients is higher than that compared with normal tissues. Even with these elaborate findings, whether *ASPM* is involved in the malignant transformation of hepatitis B or cirrhosis and the precise role it plays in this process remains unclear. Recently, our group demonstrated that *ASPM* was highly expressed both in hepatitis B virus (HBV) ^(+)^ HCC and HBV ^(+)^ non-cancerous expression profiles, and with the progression of hepatitis B cirrhosis, *ASPM* showed significant genetic alteration potential and overexpression tendency, suggesting that *ASPM* might involve in the malignant progression of hepatitis B cirrhosis. Also, *ASPM* was associated with poor prognosis of HCC patients and was highly correlated with the level of immune cell infiltration. The workflow of the present study was shown in Figure 1A.

## Identification of Hub Genes in HBV-Positive HCC

To examine the biological pathways and genes involved in HBV-associated HCC, GSE121248, GSE84402, and GSE55092 gene expression profiles, including 70, 13, and 49 cases of HBV ^(+)^ HCC liver tissue/hepatocyte, and 37, 13, and 91cases of HBV ^(+)^ non-cancerous liver tissue/hepatocyte respectively, were retrieved from the Gene Expression Omnibus (GEO) database. Overall, 295 downregulated and 134 upregulated differentially expressed genes (DEGs) were identified by using the GEO2R tool (Supplementary Figure S1; Supplementary Table S1). Functional enrichment analysis of the DEGs uncovered that upregulated genes functioned in cell cycle, DNA replication, and p53 pathways, suggesting that the 134 upregulated DEGs were critical to the development of HCC (Supplementary Figure S2). Visualization of the protein-protein interaction (PPI) network of 134 DEGs was done using the online tool, STRING database (Supplementary Figure S3A). Then, Cytoscape software was utilized to construct PPI networks. The module containing 70 genes was identified as the most significant by MCODE with max depth = 100, k-core = 2, node score cutoff = 0.2, and degree cutoff = 2 (Supplementary Figure S3B). Finally, all 22 hub genes with the same highest connectivity were chosen through Maximal Clique Centrality (MCC) and Degree algorithm from the module (Figure 1B; Supplementary Table S2). Biological process and pathway analysis revealed that the 22 hub genes, including *ASPM*, *BUB1*, *BUB1B*, *CCNA2*, *CCNB1*, *CCNB2*, *CDK1*, *CENPF*, *DLGAP5*, *KIF1*, *KIF20A*, *KIF23*, *KIF2C*, *KIF4A*, *MAD2L1*, *MELK*, *NCAPG*, *NUSAP1*, *PBK*, *TOP2A*, *TPX2*, and *TTK*, were specifically enriched in the regulation of nuclear division, cell cycle, p53 signaling pathway (Figure 1C). GEPIA analysis revealed that 20 of the 22 hub genes had higher expression levels in HCC samples than those in normal liver samples (*p* < 0.05, Supplementary Figure S4). *KIF11* and *TTK* had similar expression trends with other hub genes, but there was no statistical significance in HCC patients compared with normal people based on the TCGA database (*p* ≥ 0.05, Supplementary Figure S4). In addition, Overall survival analysis using the Kaplan Meier Plotter database showed that the higher the expression level of these 22 hub genes, the worse the prognosis of HCC patients (*p* < 0.05, Supplementary Figure S5).

## *ASPM* May be Related to the Malignant Progression of Hepatitis B

Subsequently, we employed cBioPortal to explore the genetic changes in the hub genes. The alterations include missense mutation, amplification, deep deletion, etc. Figure 1D showed information regarding the alterations in the 22 hub genes. Of the 353 queried patients/samples, 103 (29%) had hub gene genetic alterations, among which *ASPM* and *CENPF* changed most frequently (both 8%). Utilizing the UCSC Cancer Genomics Browser, hierarchical clustering of hub genes indicated that the expression levels of *ASPM* were higher in the HCC samples (Figure 1E). Using GENEVESTIGATOR software, 14 disturbances were selected from the HS_AFFY_U133PLUS database to explore the changes in the expression level of hub genes in different tissues (Figure 1F). According to the results, we found that the expression level of *ASPM* in liver cancer tissues was much higher in comparison to the normal tissues, and the level of expression increased with the progression of HCC. In the early stage of HCC, the *ASPM* expression levels were also increased, and the closer to the tumor center, the higher the level of its expression. We then examined the hub gene expression level in liver fibrosis tissue. More significantly, the results showed that there were some genes such as *ASPM, NUSAP1,* and *PBK* showed increased expression levels in non-tumor tissues with advanced liver fibrosis (Figure 1G). Also, the results of hierarchical clustering (based on the UCSC database) indicated that the expression levels of these genes increased with the progression of liver fibrosis (Figure 1H). To verify these findings, the other two datasets (GSE118295 and GSE69590, both contained three pairs of HBV-positive and normal hepatocyte samples) were retrieved from the GEO database, and *ASPM* was considered to be the most promising biomarker because it also showed an up-regulation trend as DEG (|logFC|>1 and *p* < 0.05 were set as the cut-off criteria) in HBV-infected hepatocytes compared to normal liver cells (Supplementary Figure S6). In addition, based on the results of the GEPIA and Kaplan and Meier Plotter database, we found that *ASPM* was not only statistically highly expressed in HCC (Figure 1I), but was also associated with significantly worse survival in HCC patients (Figure 1J). These results suggested that the expression of *ASPM* might be abnormal in the stage of hepatitis B cirrhosis prior to tumor development, which reminded us that *ASPM* might play a vital function in the progression of hepatitis B cirrhosis into HCC.

Based on the TCGA database, we compared the expression levels of ASPM in HBV (+) HCC and HBV (-) HCC patients, as well as in HCV (+) HCC and HCV (-) HCC patients. Compared with adjacent non-tumor tissues, the expression level of *ASPM* in HBV (+) HCC tissues (*n* = 145) was significantly higher than that in HBV (-) HCC tissues (*n* = 226). However, there was no significant difference in the expression level of *ASPM* in HCC tissues regardless of HCV infection (Figures 1K,M). In addition, we further performed the prognostic analysis in HBV (+) and HBV (-) HCC patients, as well as HCV (+) and HCV (-) HCC populations. Results showed that the prognosis of HCC patients infected with HBV or HCV was significantly worse than that of HBV or HCV-negative patients (Figures 1L,N). Meanwhile, as shown in Figure 1J, HCC patients with high *ASPM* expression had a worse prognosis than those with lower *ASPM* expression. These results suggested that the increased expression of *ASPM* may be related to the poor prognosis of HBV-positive HCC patients. However, the correlation between *ASPM* expression level and prognosis in HBV-positive patients remains to be further studied.

## Immune Infiltration Analysis, miRNA Prediction, Pan-Cancer Analysis, Co-Expression Gene Prediction, and Pathway Correlation Analysis Were Performed for *ASPM*

**FIGURE 2:**
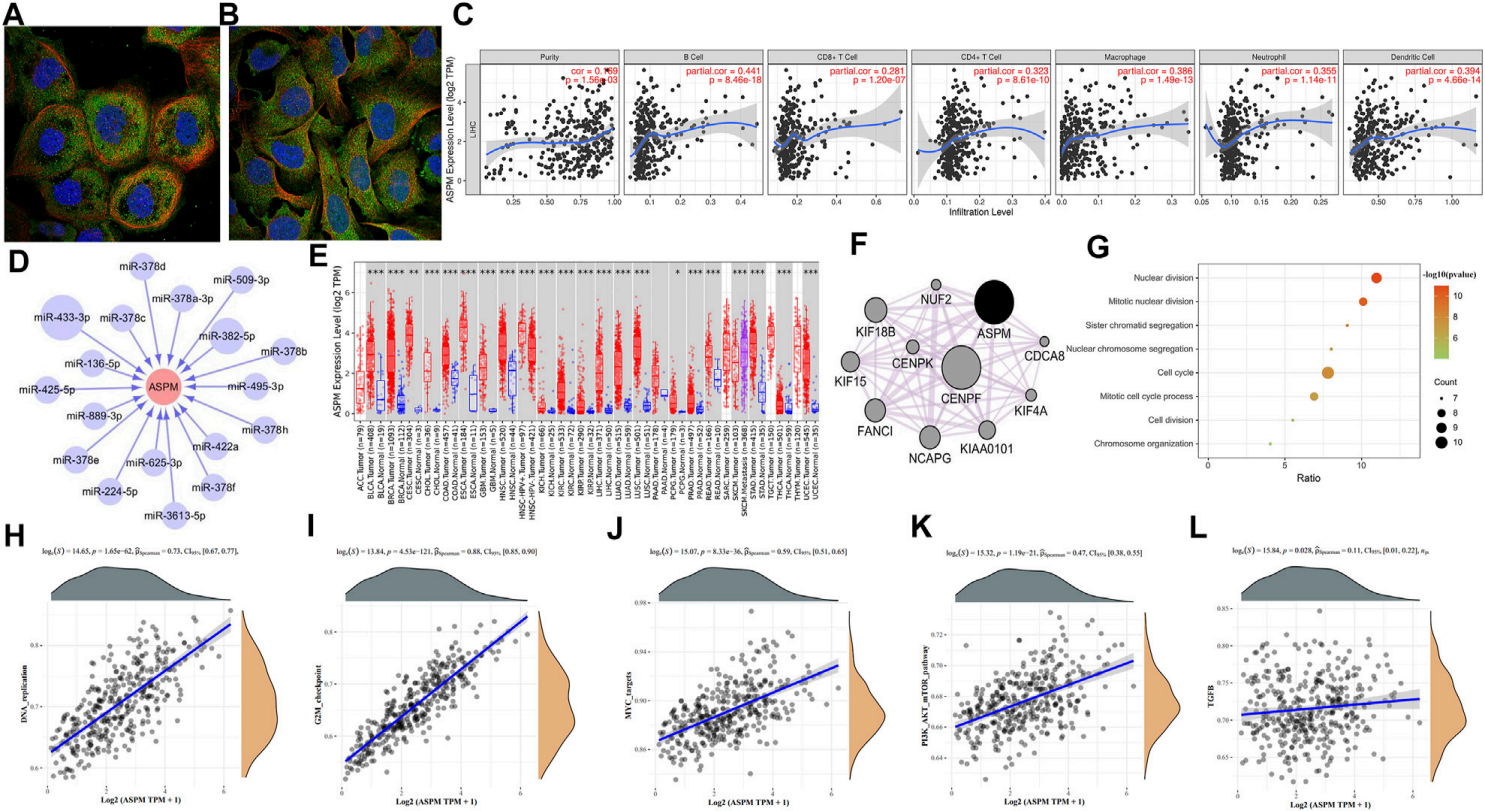
**(A)** Fluorescence staining of *ASPM* in A-431 cells was obtained from Human Protein Atlas database. **(B)** Fluorescence staining of *ASPM* in U-2 OS cells was obtained from Human Protein Atlas database. **(C)** Correlation analysis of *ASPM* expression and immune infiltration evaluated by the purity-corrected partial Spearman’s rho test. **(D)** miRNA-gene interaction network constructed based on PITA, miRanda, TargetScan, and PicTar database. **(E)** *ASPM* expression in human normal and tumor tissues were statistically evaluated by the Wilcoxon test **(F)** The PPI network of *ASPM* and its co-expression genes was created by GeneMANIA software in Cytoscape. **(G)** Biological process analysis of *ASPM* co-expressed genes **(H–L)** The correlation between *ASPM* and pathway was explored using R software GSVA package and was analyzed by Spearman correlation; the X-axis represents gene expression, the Y-axis represents the pathway score, and the density curve on the right represents the distribution trend of the pathway score; the density curve on the upper side represents the distribution trend of gene expression; the uppermost value represents the correlation *p*-value, correlation coefficient, and correlation calculation method. *: *p*-value < 0.05; **: *p*-value <0.01; ***: *p*-value <0.001.

Data retrieved from the Human Protein Atlas database represent that *ASPM* was mainly located in the plasma membrane or cytosol (Figures 2A,B). As reported by a large number of reports, immune cells can induce the remodeling of the immune microenvironment of the tumor, thus affecting the prognosis of patients with HCC. Correlation analysis based on Tumor Immune Estimation Resource database showed that *ASPM* was related to tumor immune cell infiltration, and the infiltration levels of B cells, CD8^+^ T cells, CD4^+^ T cells, macrophages, neutrophils, and dendritic cells were all positively correlated with the expression of *ASPM* (Figure 2C). We next explored the regulatory associations between *ASPM* and miRNA, four miRNA target prediction databases including PITA, miRanda, TargetScan, and PicTar were employed to predict the miRNA targets of hub genes. miRNAs predicted by at least two databases were chosen as the target miRNAs of the hub gene. The interaction between the gene and the miRNA was represented by an arrow, and the size of the circular node was adjusted according to the number of supporting databases (Figure 2D). In the network, miR-433-3p, supported with the greatest number of prediction databases, inhibition of which could enhance the viability, migration, and invasion of HeP3B cells (Song et al., 2021). In addition, miR-433-3p was found to be highly expressed in serum, and Tan Y, et al. demonstrated that this might help in diagnosing HBV-related HCC (Tan et al., 2014). We noticed that there were multiple members of the miR-378 family (miR-378a-3p, miR-378b, miR-378c, miR-378d, miR-378e, miR-378f, miR-378h) in the *ASPM*-targeted miRNAs in the network. Some studies have shown that members of this family are associated with inhibiting liver fibrosis or aggressive tumor behavior. For instance, in the carbon tetrachloride-induced chronic liver fibrosis mouse model, overexpression of miR-378a-3p could inhibit the activation of hepatic stellate cells and reduce the expression of pro-fibrosis genes in liver tissues (Hyun et al., 2016). Competitively sponging miR-378a-3p promoted HCC proliferation and angiogenesis (Ji et al., 2021). In addition, other studies have shown that activation of miR-378a transcription could potentiate sorafenib sensitivity in HCC, which may be related to the regulation of sorafenib-induced apoptosis of HCC cells (Lin et al., 2020). Studies have shown that a low level of miR-136–5p leads to inhibition of its interaction with target genes, thereby promoting the proliferation of HCC cells (Shiu et al., 2021). On the other hand, the elevated level of miR-382-5p in HBV-positive HCC cells could mediate the involvement of hepatitis B core protein (HBc) in promoting HCC cell metastasis (Du et al., 2018). However, the specific roles and mechanisms of miRNAs and *ASPM* in disease progression and occurrence remain to be studied. Pan-cancer analysis showed that the expression of *ASPM* increased in many cancers type such as lung adenocarcinoma (LUAD) and breast cancer (BRCA), which indicates that more significance of research value for *ASPM* needs to be explored for further study (Figure 2E). Figure 2F was the network of *ASPM* and its 10 most relevant co-expressed genes constructed by the GeneMANIA plug-in in Cytoscape software. Interestingly, centromere protein F (*CENPF*), as the gene most closely related to the co-expression of *ASPM*, was one of the 22 hub genes screened based on sequencing data, and *CENPF* and *ASPM* both have the same 8% genetic alterations. The results of expression verification and prognostic analysis showed that *CENPF* was highly expressed in HCC tissues too (Supplementary Figure S4), and the high expression of which was associated with poor prognosis of HCC patients (Supplementary Figure S5). Studies have shown that activating transcription of *CENPF* could promote tumor growth of HCC, and clinically, overexpression of *CENPF* correlated with shorter overall survival and higher cumulative recurrence rates of HCC (Yang et al., 2019). *CENPF* may also be a novel predictor for prognosis and a potential therapeutic target for HCC. Functional enrichment analysis of *ASPM* co-expressed genes showed that these genes were enriched in the nuclear division, mitotic nuclear division, sister chromatid segregation, nuclear chromosome segregation, cell cycle, mitotic cell cycle process, cell division, and chromosome organization (Figure 2G). To study the correlation between *ASPM* and tumor-related pathways, the R software GSVA package was used to analyze method = “ssgseaˮ. The correlation between genes and pathway scores was analyzed by Spearman correlation. We found that *ASPM* was significantly correlated with DNA replication, PI3K-AKT-mTOR pathway, G2M checkpoint, transforming growth factor-β (TGFB) pathways (Figures 2H–L). It is of great research significance to explore the involvement of *ASPM* in specific pathophysiological processes through these pathways.

In this study, there were still several limitations worth noting. Firstly, we mainly discussed the probable roles and functions of hub genes but did not conduct an in-depth analysis of other DEGs. Further research in this area is needed in the future. Secondly, this study employed only TCGA, GEPIA, and GENEVESTIGATOR databases to confirm the expression level of hub genes. As such, there is a need to conduct further studies to validate the findings of the current study. Lastly, because of limited data, the clinical data of HBV-related HCC patients were not analyzed in depth. Nevertheless, our study provides new insights into HBV-related HCC. Integrated bioinformatics analysis may provide more accurate results than a single dataset study. In addition, the potential treatment targets and candidate drugs reported herein have a broad application prospect in individualized therapy. Moreover, the established miRNA-hub gene network may reveal the significance of epigenetic regulation in HBV-associated HCC.

In conclusion, this study conducted a comprehensive bioinformatics analysis of DEGs in HBV-positive HCC and identified 22 hub genes that may play an essential role in the development and progression of HBV-associated HCC. The expression of *ASPM* was increased in both the advanced stage of hepatitis B fibrosis and the early stage of HCC and is considered as a potential biomarker for HBV-associated HCC. Further studies are still needed to explore the biological function of *ASPM* in the pathogenesis of HBV-associated HCC.

## Data Availability

The original contributions presented in the study are included in the article/Supplementary Material, further inquiries can be directed to the corresponding author.
